# Characteristics of Breast Cancer Metastasizing to Bone in a Mediterranean Population

**DOI:** 10.7759/cureus.11679

**Published:** 2020-11-24

**Authors:** Sami Bannoura, Hasan Nahouli, Aya Noubani, Abdallah Flaifel, Ibrahim Khalifeh

**Affiliations:** 1 Pathology, American University of Beirut Medical Center, Beirut, LBN; 2 Orthopaedic Surgery, American University of Beirut Medical Center, Beirut, LBN; 3 Global Health Institute, American University of Beirut, Beirut, LBN

**Keywords:** breast cancer, bone metastasis, middle east

## Abstract

Aim: This study examines clinicopathological, molecular, and radiological characteristics of breast cancer metastasizing to the bone in a Mediterranean population.

Methods: Cases of breast cancer with metastasis to bone were retrieved from the pathology department archives. Descriptive statistics and bivariate inferential statistics of retrieved clinical (demographic, focality, laterality, axillary lymph node status, and metastasis-free interval), radiological (skeletal site of bone metastasis, type of bone lesion), and microscopic (grade, subtype of breast cancer, lymphovascular status, perineural status, lymph node involvement, nodal extracapsular extension, molecular subtype) data were conducted.

Results: Out of 123 cases analyzed, 93.5% were ductal, 90% had axillary lymph node metastasis, 60.5% were luminal A, 59.6% were osteolytic, and 54.4% had grade III. Discordance in the status of ER, PR, and HER2 between the primary breast tumor and the corresponding bone metastases was noted, with the highest rate of change reported for PR (35.7%). Significance was detected at the level of difference between the subtype of breast cancer with regards to the radiologic features where the ductal subtype was found to be mostly osteolytic while the lobular subtype was mostly either osteoblastic or mixed (p-value=0.05). The metastasis-free interval was significantly associated with the number of metastatic bone lesions (P=0.001).

Conclusion: The significant association between metastasis-free interval and the number of metastatic bone lesions suggests that a higher interval allows more time for tumors to manifest multiple lesions. The high rate of discordance in the status of PR, ER, and HER2 was congruent with the literature highlighting the need to further investigate underlying mechanisms.

## Introduction

Breast cancer is the most common female malignancy and the primary cause of death among women worldwide. The incidence rate of breast cancer among Lebanese women is projected to reach 146.1 cases per 100,000 females by 2020, accounting for 40.4% of all cancers among women [1]. Studies show that the burden of breast cancer in Lebanon is higher than that of countries in the region [2].

Breast cancer can be categorized based on different criteria including histologic type, expression of tumor markers, and clinical features [3]. The most frequent histologic types of breast cancer are ductal and lobular carcinomas, with the ductal type being the most prevalent [3,4]. At the clinical level, the different prognostic features of breast cancer comprise the age at diagnosis, tumor size, grade, lymphovascular invasion, surgical margins, menopausal status, as well as lymph nodes, and distant metastasis. At the molecular level, four subgroups of breast cancers exist based on estrogen (ER) and progesterone [5] gene heterogeneity [5]: luminal A (ER or PR+, Human Epidermal Growth Factor Receptor 2- (HER2-)), luminal B (ER- or PR+, HER2+), ER-PR-HER2+, and triple-negative (ER-PR-HER2-) types.

Breast cancer progression often includes metastasis, which is the spread and development of tumor cells in distant organs [6]. Most women with advanced breast cancer develop bone metastases [6,7]. Based on the radiological appearance, metastasis of breast cancer to the bone can cause mostly two types of bone metastatic lesions: osteolytic and osteoblastic. A mixed lytic-blastic appearance can be observed as well. Hypotheses about the metastasis of breast cancer cells to the bone being driven by oncogenic mutations that exist in cancerous cells of the primary tumor are widely proposed in the literature [6]. Kang et al. suggest that a set of transforming events followed by an accumulation of oncogenic genetic and epigenetic alterations dynamically interfere with the development of the primary breast tumor and drives its metastasis to bone [6].

The aim of this study is to examine the clinicopathological, molecular, and radiological characteristics of breast cancer metastasizing to the bone in patients who presented at a tertiary healthcare center in the Eastern Mediterranean region.

## Materials and methods

Case selection

This is a retrospective cohort study where a review of the archives of the Pathology Department at the American University of Beirut Medical Center (AUBMC) between the years 1996 and 2016 was conducted. A dataset of breast cancer patients presenting with biopsy-confirmed bone metastasis was created. 123 cases were identified and included in the study. No personal identiﬁers were used. Cases with incomplete clinical information were excluded.

Clinicopathologic data

Multiple clinical variables were retrieved from the medical records including demographics (e.g., age and gender), focality and laterality of the tumor, axillary lymph node status, stage (according to AJCC 8th edition) [8], and the metastasis-free interval which represents the interval between the first diagnosis of breast cancer and the time of first presentation with metastasis.

Radiological data

Radiological information was collected from the reviewed records with available different imaging modalities. The skeletal site of bone metastasis was recorded as involving the axial, appendicular skeletons, or both. Axial skeletal sites consisted of the cranium, face, vertebrae, ribs, or sternum, while the appendicular skeletal sites included the pelvis, upper, and lower extremities. The type of bone lesion was collected and categorized as osteolytic, osteoblastic, or mixed. The number of lesions was noted.

Microscopic data and predictive markers

This included the recording of the subtype of breast cancer (ductal, lobular, carcinoma, mixed ductal, and lobular), tumor grade (I, II, III), the presence or absence of lymphovascular invasion, perineural invasion, lymph node involvement, and nodal extracapsular extension. Data on predictive markers of both the primary and metastatic tumors were also collected. These included ER, PR, and HER2. In addition, the molecular subtype (luminal A, luminal B, triple negative, HER2 amplified) was recorded for both the primary breast tumor and the bone metastasis in each patient.

Statistical analysis

Descriptive statistics were conducted for the different variables included in the study. The results for the categorical variables were reported as frequencies and percentages, while the mean and standard deviation was reported for the continuous one (metastasis-free interval). The shapiro wilk test was conducted to test the normality of the continuous dependant variable (metastasis-free interval) having a p-value = 0.106. Bivariate inferential statistics were conducted to determine any significant associations and correlation between the independent variables (focality/ centricity, primary grade, number of lesions, primary and metastatic molecular subtypes, subtypes of breast cancer, axillary lymph nodes status) and the dependant ones (metastasis-free interval and radiologic features). Chi-square test and Fischer exact test were used for the categorical and binary factors, while independent t-test and one-way ANOVA test were conducted for the continuous variable (metastasis-free interval). The results were presented in the tables by the p-values, in addition to the descriptive results for each of the dependent groups defined (metastasis-free interval and radiologic features). The data analysis was conducted on SPSS 22 (IBM Corp., Armonk, NY). Significance was determined by p-value < 0.05.

## Results

Clinicopathologic variables

Out of the 123 included breast cancer cases metastasizing to the bone, the majority of the patients were females (98.4%), with a mean age of 52.5±14.2 years. The mean metastasis-free interval was 61.37±48.67 months. Around 17% of the patients presented with a zero metastasis-free interval underlining metastatic bone lesions upon presentation. Most of the breast cancer cases were unilateral (95.9%), and the majority were unifocal (88.6%). The highest proportion of patients was in the N1a axillary lymph node stage group (15.4%; Table 1).

**Table 1:** Descriptive characteristics of the tumor markers and the cytological features of the included breast cancer cases SD: standard deviation, HER2: human epidermal growth factor receptor 2, ER: estrogen, PR: progesterone.

Variable		N (%)	Variable		N (%)
Clinical characteristics					
Gender	Male	2 (1.6%)	Laterality	Unilateral	118 (95.9%)
	Female	121 (98.4%)		Bilateral	5 (4.1%)
Age (year)	Mean ± SD	52.5 ± 14.2			
Axillary lymph node status	Negative nodes	5 (4.1%)	Focality/centricity	Unifocal	109 (88.6%)
	N1a	19( 15.4%)		Multifocal	14 (11.4%)
	N2a	3 (2.4%)	Metastasis-free interval	Mean ± SD (months)	61.37 ± 48.67
	N3a	4 (3.3%)			
	Not available	92 (74.8%)			
Microscopic and molecular characteristics					
Primary ER	Negative	8 (17.0%)	Metastatic ER	Negative	30 (27.8 %)
	Positive	39 (83.0%)		Positive	78 (72.2%)
Primary PR	Negative	13 (27.7%)	Metastatic PR	Negative	57 (53.3%)
	Positive	34 (72.3%)		Positive	50 (46.7%)
Primary HER2	Negative	36 (81.8%)	Metastatic HER2	Negative	91 (90.1%)
	Positive	8 (18.2%)		Positive	10 (9.9%)
Metastatic molecular subtype	Luminal A	75 (61.0%)	Primary molecular subtype	Luminal A	36 (29.3%)
	Luminal B	3 (2.4%)		Luminal B	4 (3.2%)
	Triple-negative	16 (13.0%)		Triple-negative	1 (0.9%)
	HER2 amplified	7 (5.7%)		HER2 amplified	4 (3.2%)
	Missing/Not available	22 (17.9%)		Missing/not available	78 (63.4%)
Perineural invasion	Not identified	119 (96.7%)	A subtype of breast cancer	Ductal	115 (93.5%)
	Present	4 (3.3%)		Lobular	6 (4.9%)
				Sarcoma	1 (0.8%)
Lymphovascular invasion	Not identified	111 (90.2%)		Mixed ductal and lobular	1(0.8%)
	Present	12 (9.8%)			
Extracapsular extension	Absent	114 (92.7%)	Primary grade	I	4 (9.2%)
	Present	9 (7.3%)		II	16 (36.4%)
				III	24 (54.4%)
Radiologic characteristics					
Skeletal distribution of lesions by imaging	Appendicular	9 (7.3%)	Number of lesions	Not available	35 (28.4%)
	Axial	27 (21.9%)		One	21 (17.1%)
	Combination	51 (41.5%)		Two or more	67 (54.5%)
	Not available	36 (29.3%)			
Radiologic features	Osteolytic	59 (59.6%)			
	Osteoblastic	28 (28.3%)			
	Mixed	12 (12.1%)			

Radiologic variables

The majority of the cases had a combination of appendicular and axial skeletal involvement by metastatic lesions (41.5%) and 59.6% of them were osteolytic (Table 1).

Microscopic and predictive Markers Variables

Analysis of the primary breast cancer in Table 1 showed that most of the included breast cancer cases were of ductal type (93.5%), and the majority were of high grade (54.4%). For the primary breast tumor, ER was positive in 83%, PR was positive in 72.3%, while 18.2% of cases had HER2 overexpression. The associated bone metastasis was positive for ER and PR in 72.2% and 46.7% of cases, respectively. Metastatic HER2 was overexpressed in 5.7% of cases. In the case of metastasis, the dominant subtype was the luminal A (61.0%), followed by triple-negative (13.0%), and HER2 amplified (5.7%). A remarkable change in hormonal and HER2 status was noticed between primary and metastatic tumors (Table 2). The highest rate of discordance was noted in PR (35.7%), followed by ER (21.5%), and HER2 (15.4%).

**Table 2:** The frequency of change in each of the three tumor markers: estrogen, progesterone, and HER2 ER: estrogen, PR: progesterone, HER2: human epidermal growth factor receptor 2.

ER change	N (%)	PR change	N (%)	HER2 change	N (%)
No change	33 (78.6%)	No change	27 (64.3%)	No change	33 (84.6%)
Positive-negative change	7 (16.6%)	Positive-negative change	12 (28.6%)	Positive-negative change	5 (12.8%)
Negative-positive change	2 (4.8%)	Negative-positive change	3 (7.1%)	Negative-positive change	1 (2.6%)

The bivariate associations between the included variables and the dependent groups (metastasis-free interval and radiologic features) were examined (Table 3). The mean time (in months) of the metastasis-free interval to tissue diagnosis was significantly different in the case of the number of metastatic bone lesions. Patients with two or more bone lesions had a higher mean of metastasis-free interval than those with one lesion (p-value=0.001).

**Table 3:** The bivariate associations between the presented variables and the two dependent groups: metastasis-free interval and radiologic features were examined SD: standard deviation, HER2: human epidermal growth factor receptor 2, ER: estrogen, PR: progesterone. *Statistical Significance with p-value<0.05

Variable		Metastasis-free interval (months)	P-value	Radiologic features			P-value
Clinical variables							
		Mean/ SD		Osteolytic	Osteoblastic	Mixed	
Focality/centricity	Unifocal	60.9±50.1	0.799	55 (62.5%)	24 (27.3%)	9 (10.2%)	0.14
	Multifocal	57.3±39.4		4 (36.4%)	4 (36.4%)	3 (27.3%)	
Axillary lymph node status	Negative nodes	46.6± 30.7	0.72	1 (20.0%)	3(60.0%)	1 (20.0%)	
	Others	54.0±45.1		14 (63.6%)	4 (18.2%)	4 (18.2%)	
Molecular variables							
Primary grade	I	51.0±48.3	0.59	2 (50.0%)	0 (0.0%)	2 (50.0%)	0.07
	II and III	39.4±41.0		19 (61.3%)	10 (32.3%)	2 (6.5%)	
Met molecular subtype	Luminal A	58.1±50.7	0.24	39 (60.9%)	17 (26.6%)	8 (12.5%)	0.23
	Luminal B	48.0±24.0		1 (33.3%)	2 (66.7%)	0 (0.0%)	
	Triple-negative	55.4±40.8		11 (84.6%)	1 (7.7%)	1 (7.7%)	
	HER2 amplified	48.3±38.0		4 (57.1%)	2 (28.6%)	1 (14.3%)	
Subtypes of breast cancer	Ductal	59.8±48.4	0.32	57 (62.0%)	26 (28.3%)	9 (9.8%)	0.05*
	Lobular	71.0±55.5		1 (20.0%)	2 (40.0%)	2 (40.0%)	
Primary ER	Negative	63.8±68.1	0.38	4 (66.7%)	2 (33.3%)	0 (0.0%)	0.84
	Positive	40.9±38.5		17 (53.1%)	10 (31.2%)	5 (15.6%)	
Primary PR	Negative	52.2±60.3	0.57	6 (60.0%)	3 (30.0%)	1 (10.0%)	1
	Positive	41.9±38.0		15 (53.6%)	9 (32.1%)	4 (14.3%)	
Primary HER2	Negative	44.61±42.9	0.78	18 (62.1%)	6 (20.7%)	5 (17.2%)	0.21
	Positive	39.88±47.3		3 (42.9%)	4 (57.1%)	0 (0.0%)	
Primary molecular subtype	Luminal A	41.00± 38.4	0.38	17 (60.7%)	7 (25.0%)	4 (14.3%)	0.57
	Luminal B	45.25±57.2		1 (25.0%)	3 (75.0%)	0 (0.0%)	
	Triple-negative	110.50±98.3		1 (100.0%)	0 (0.0%)	0 (0.0%)	
	HER2 amplified	35.50±43.2		2 (66.7%)	1 (33.3%)	0 (0.0%)	
Radiologic variables							
Number of lesions	One	31.2±37.6	0.001*	13 (59.1%)	6 (27.3%)	3 (13.6%)	0.76
	Two or more	64.9±48.7		36 (56.2%)	19 (29.7%)	9 (14.1%)	

Significance was also detected at the level of difference between the subtype of breast cancer in regards to the radiologic features where the ductal subtype was found to be mostly osteolytic while the lobular subtype was mostly either osteoblastic or mixed (p-value=0.05).

## Discussion

Several studies showed that, following lymph nodes, bone is the most common site of distant metastasis in breast cancer [9,10] which highlights the importance of this study examining the clinicopathological, molecular, and radiological characteristics of breast cancer metastasizing to the bone.

Clinicopathologic variables

Literature suggests the importance of the metastasis-free interval as a prognostic factor [10,11], where a short metastasis-free interval suggests a more aggressive, rapidly growing tumor. James et al. also found that low-grade and ER+ tumors showed significantly prolonged survival and thus longer metastasis-free intervals, compared to patients with high-grade or ER-tumors [10]. In contrast, the bivariate analysis of this study did not detect any significant difference in the mean metastasis-free interval to tissue diagnosis neither between different grade categories nor between different primary ER status. Similarly, James et al. reported that factors of less aggressive tumor phenotype such as low-grade were more likely to metastasize to bone. However, our study found that almost half of the cases were grade III.

For demographic variables, some studies suggest that age does not play a role in the metastasis of breast cancer to the bone where bone metastasis appears to be evenly distributed across different age groups [10]. Other studies, on the other hand, report that older women are more likely to perceive bone metastasis [12].

In this study, the mean age of included patients with breast cancer metastasizing to bone was 52.5±14.2 years, identical to the mean age found by Salem et al. among breast cancer patients in Lebanon [13].

Radiologic variables

In regards to the radiographic appearances of the bone metastases, some studies have found that it is not associated with grade, ER status, or the type of primary tumor [10]. This is consistent with the findings of our study where none of the variables showed a significant difference between the three categories of the bone radiologic features except for the ductal subtype of breast cancer, which showed a significant relationship with osteolytic bone lesions. In contrast, James et al. found no relationship between the radiographic appearance of the bone metastases and the type of primary tumor [10].

Microscopic and predictive markers variables

At the microscopic level, studies report ER+ breast tumors highly metastasize to bone [10,14-16], similar to our findings where the primary ER tumor marker was positive in 83.0% of cases.

Regarding the molecular subtype, findings from this study showed that the dominant subtype was luminal A (80.0%), while the least common was triple-negative (2.2%). This is in contrast to findings from a study by Kennecke et al. on the metastatic behavior of breast cancer subtypes, which reported bone as the predominant site of metastasis for Luminal B (71.4%) [17]. Yet, another finding of the same study by Kennecke et al. is consistent with our results, indicating that triple-negative tumors had a significantly low rate of bone metastasis compared to luminal A [17].

As for the significant association between metastasis-free interval and the number of bone lesions, it suggests that a higher interval allows more time for tumors to manifest multiple metastatic bone lesions, i.e., the burden of bone metastatic lesions is a matter of time. Similarly, the result of triple-negative subtype having a significantly longer metastasis-free interval compared to other subtypes could be potentially explained by a finding from literature reporting that triple-negative tumors are highly metastatic to brain, lung, and to a significantly lesser extent to the bone [17]. Thus, a longer metastasis-free interval is needed for a triple-negative tumor to be detected as metastasizing to bone due to prior involvement in metastasis to other organs.

In regards to the breast cancer subtype, several previous studies found no evidence that lobular carcinomas are more likely to metastasize to bone compared to the ductal subtype [18,19]. Other studies suggest that the lobular subtype is more likely to metastasize to bone [9,20]. On the contrary, we found in this study that most of the included breast cancer cases metastasizing to bone were of ductal subtype (93.5%). This is consistent with our unpublished data on review of the complete archives of the breast cancer cases from 1996 till present at the American University of Beirut Medical Center showing a proportional rate of ductal (90%) to lobular (10%) subtype. Similarly, studies from the literature suggest congruent figures with the most invasive breast cancer carcinomas being of the ductal type and only about 10% of invasive lobular type [21,22].

Discordance in the status of tumor markers

As for the change in the status of receptors, discordance in the status of ER, PR, and HER2 between the primary tumor and subsequent metastases is well reported in the literature [5,23]. Different studies reported different rates of change in single-receptor expression [23,24] with the highest rate of change reported for PR. This is consistent with our study where PR was the receptor that exhibited a higher rate of status change. More specifically, studies indicate that PR loss was the main change observed [25-28], a finding that is congruent with the results of our study in which the most prevalent change of status in discordant cases was the change of PR from positive to negative. However, among the HER2 discordant cases, our study showed that more patients lost the HER2 expression than those who became HER2-overexpressed, which contradicts the findings of a meta-analysis highlighting contrary results [29].

Literature suggests that the mechanisms of switching are still unknown and could have both biological and therapeutic implications. There are several possible explanations for these changes such as technical issues (e.g., variability in assay performance), biological mechanisms (biologic evolution of the tumor), and tumor heterogeneity [23,30].

Limitations

The main focus of this study is on patients presenting only with bone metastasis due to breast cancer in a limited geographic area which may be a source of bias. Additionally, concomitant visceral metastases were not included in our study sample in which may contribute better to our understanding of the metastatic patterns for different molecular subtypes of breast cancer. Finally, the inclusion of a larger number of cases would have been more informative in our study. 

## Conclusions

This study examined clinicopathological, molecular, and radiological characteristics of breast cancer metastasizing to the bone in patients who presented at a tertiary healthcare center in the Middle East region. The high rate of discordance in the status of PR, ER, and HER2 was congruent with the literature highlighting the need to further investigate underlying mechanisms. The significant association between metastasis-free interval and the number of metastatic bone lesions underlines the need for further research to explore the nature of the correlation between these two variables with the suggestion that a higher interval allows more time for tumors to manifest multiple lesions being a potential explanation.
